# Prevention of Paclitaxel-induced allodynia by Minocycline: Effect on loss of peripheral nerve fibers and infiltration of macrophages in rats

**DOI:** 10.1186/1744-8069-6-76

**Published:** 2010-11-05

**Authors:** Cui-Cui Liu, Ning Lu, Yu Cui, Tao Yang, Zhi-Qi Zhao, Wen-Jun Xin, Xian-Guo Liu

**Affiliations:** 1 Department of Physiology and Pain Research Center, Zhongshan Medical School, Sun Yat-Sen University, 74 Zhongshan Rd. 2, Guangzhou, 510080, PR China; 2 Institute of Neurobiology, Institutes of Brain Science and State Key Laboratory of Medical Neurobiology, Fudan University, Shanghai 200032, PR China

## Abstract

**Background:**

Although paclitaxel is a frontline antineoplastic agent for treatment of solid tumors, the paclitaxel-evoked pain syndrome is a serious problem for patients. There is currently no valid drug to prevent or treat the paclitaxel-induced allodynia, partly due to lack of understanding regarding the cellular mechanism. Studies have shown that minocycline, an inhibitor of microglia/macrophage, prevented neuropathic pain and promoted neuronal survival in animal models of neurodegenerative disease. Recently, Cata *et al *also reported that minocycline inhibited allodynia induced by low-dose paclitaxel (2 mg/kg) in rats, but the mechanism is still unclear.

**Results:**

Here, we investigate by immunohistochemistry the change of intraepidermal nerve fiber (IENF) in the hind paw glabrous skin, expression of macrophage and activating transcription factor 3 (ATF3) in DRG at different time points after moderate-dose paclitaxel treatment (cumulative dose 24 mg/kg; 3 × 8 mg/kg) in rats. Moreover, we observe the effect of minocycline on the IENF, macrophages and ATF3. The results showed that moderate-dose paclitaxel induced a persisted, gradual mechanical allodynia, which was accompanied by the loss of IENF in the hind paw glabrous skin and up-regulation of macrophages and ATF3 in DRG in rats. The expressions of ATF3 mainly focus on the NF200-positive cells. More importantly, we observed that pretreatment of minocycline at dose of 30 mg/kg or 50 mg/kg, but not 5 mg/kg, prevented paclitaxel-evoked allodynia. The evidence from immunohistochemistry showed that 30 mg/kg minocycline rescued the degeneration of IENF, attenuated infiltration of macrophages and up-regulation of ATF3 induced by paclitaxel treatment in rats.

**Conclusions:**

Minocycline prevents paclitaxel-evoked allodynia, likely due to its inhibition on loss of IENF, infiltration of macrophages and up-regulation of ATF3 in rats. The finding might provide potential target for preventing paclitaxel-induced neuropathic pain.

## Background

Clinical and animal research have shown that paclitaxel, a widely used chemotherapeutic agent against solid tumors, can induce a dose-dependent peripheral sensory neuropathy [1,2]. Subjects following application of paclitaxel mainly experience tingling and allodynia that often occur in a "glove and stocking" distribution [3]. The anti-tumor action of paclitaxel was due to their binding toβ-tubulin of microtubules. It has been thought that such binding impaired axoplasmic transport, thereby leading to a progressive, dying-back axonopathy [4]. Moreover, Siau *et al *reported that application of low-dose paclitaxel (2 mg/kg) induced the loss of intraepidermal nerve fibers (IENF) on day 31 after the first injection [5]. Although quantification of IENF is potentially an important tool to assess the occurrence and severity of neuropathy [6], the correlation between the loss of IENF and painful neuropathy induced by paclitaxel remains unclear.

There are no well-established treatments to prevent or minimize paclitaxel-induced neuropathic pain because of lack of cellular mechanism. Many factors such as generation of radicals [7], abnormal functions of calcium channel [8] and transient receptor potential vanilloid 4 (TRPV4) [9] have been reported to be attributed to the development of paclitaxel-induced neuropathic pain. Recently, researchers find that paclitaxel also exerts effects on the immune system and displays immunomodulatory traits [10]. For example, paclitaxel can led to infiltration of macrophages in DRG and microglia activation in spinal dorsal horn [11]. In addition, study also shows that application of minocycline, a selective microglia/macrophage inhibitor, prevents mechanical allodynia induced by paclitaxel at a low dosage of 2 mg/kg [12]. Furthermore, it has been suggested that minocycline protect the axonal dieback induced by spinal cord injury [13]. However, the mechanisms underlying the blockage of paclitaxel-induced allodynia by minocycline are still poorly understood.

Therefore, in the present study, we first observe whether moderate-dose paclitaxel (cumulative dose 24 mg/kg; 3 × 8 mg/kg, the dose was calculated from doses clinically used) could induce allodynia, reduce the density of IENF in the hind paw glabrous skin and increase expression of macrophages and ATF3 in DRG. Furthermore, we aim to elucidate whether minocycline treatment also blocks allodynia induced by moderate-dose paclitaxel. Specifically, effects of minocycline on the density of IENF in the hind paw glabrous skin, expression of cell injury marker (ATF3) and infiltration of macrophages in DRG at different time points following paclitaxel treatment are investigated.

## Result

### Minocycline prevented paclitaxel-induced mechanical allodynia

**Figure 1:**
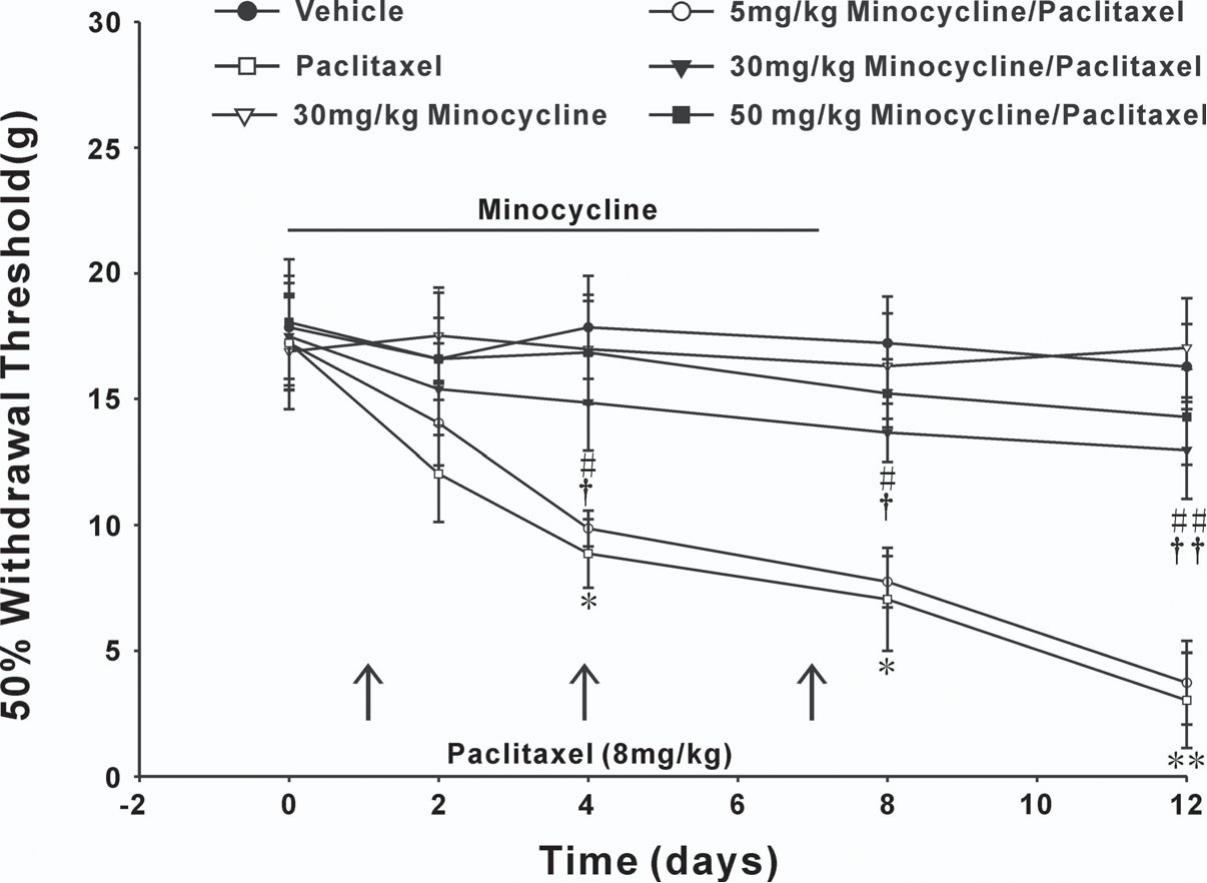
**Minocycline blocked the paclitaxel-induced mechanical allodynia**. Application of paclitaxel (cumulative dose 24 mg/kg; 3 × 8 mg/kg) induced a marked and prolonged mechanical allodynia. On day 4, 8 and 12 after first paclitaxel treatment, the paclitaxel rats showed a significant decrease in 50% withdrawal threshold relative to the vehicle group. *P < 0.05; **P < 0.01. Minocycline pretreatment at dose of 30 mg/kg or 50 mg/kg significantly attenuated paclitaxel-induced mechanical allodynia compared with the corresponding time points of paclitaxel group, respectively. # *P *< 0.05; ##*P *< 0.01 and † *P *< 0.05; †† *P *< 0.01. However, 50% withdrawal threshold had no significant difference between the 30 mg/kg minocycline/paclitaxel group and the 50 mg/kg minocycline/paclitaxel group or the vehicle group. Minocycline at 30 mg/kg alone had no effect on the mechanical withdrawal threshold in control animals (n = 8/group).

The administration of paclitaxel at a cumulative dose of 24 mg/kg (3 × 8 mg/kg, 3 days apart, i.p) caused a marked and prolonged mechanical allodynia as evidenced by 50% withdrawal threshold compared with day 0 (*P *< 0.05). On day 4 and 12 following initial paclitaxel treatment, the 50% withdrawal threshold significantly reduced to 8.87 ± 1.33 g and 3.03 ± 1.92 g respectively compared with the value (17.28 ± 1.85 g) on day 0 (Figure 1). Application of minocycline at a daily dosage of 30 mg/kg or 50 mg/kg, but not 5 mg/kg, initiated one day before paclitaxel and continued for 8 days significantly attenuated mechanical allodynia on day 4, 8 (*P *< 0.05) and 12 (*P *< 0.01) compared with the paclitaxel group. Furthermore, 50% withdrawal threshold had no significantly difference between the 30 mg/kg minocycline/paclitaxel group and the 50 mg/kg minocycline/paclitaxel group or the vehicle group. Continuous injection of minocycline at a dose of 30 mg/kg alone had no effect on mechanical hypersensivity in rats (Figure 1), so the dose of 30 mg/kg of minocycline was applied in subsequent experiments.

### Minocycline rescued the loss of IENF induced by paclitaxel

**Figure 2:**
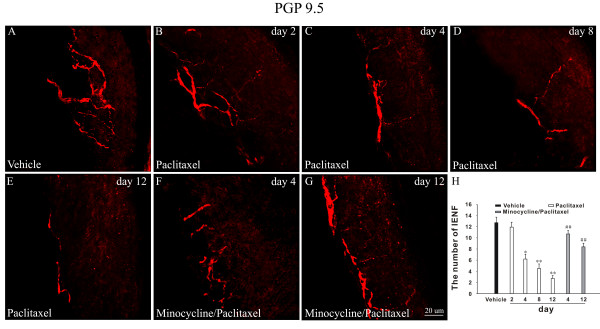
**Minocycline inhibited paclitaxel-induced loss of IENF**. PGP9.5-staining shows epidermal innervation in the hind plantar paw skin of normal rats (A). Continuous loss of PGP9.5 positive nerve fibers is evident on day 2-12 following paclitaxel treatment (B-E), *P < 0.05; **P < 0.01 compared with the vehicle group. Minocycline pretreatment attenuated the loss of IENF (F, G), ##*P *< 0.01 compared with paclitaxel group at corresponding time points. Histogram represents the mean number of IENF per sight under various treated conditions (H) (n = 5/group).

Consistent with previous studies [5], PGP9.5-labeled IENF emerged from cutaneous nerves and traveled vertically into the epidermis where they branched into terminal (Figure 2A). Following application of paclitaxel, there was a gradual decrease in the number of IENF (Figure 2H). The number of IENF per sight decreased from 12.71 ± 1 in vehicle rats to 11.92 ± 0.88, 6.2 ± 0.86, 4.5 ± 0.82 and 2.7 ± 0.53 on day 2, 4, 8 and 12 after paclitaxel treatment, respectively (Figure 2B-E). However, application of minocycline significantly inhibited the loss of IENF induced by paclitaxel on day 4 (10.69 ± 0.64) and 12 (8.58 ± 0.66) compared with that of paclitaxel group respectively (*P *< 0.01) (Figure 2F and 2G). Vehicle or minocycline alone did not affect the density of IENF compared with normal rats (data not shown).

### Minocycline inhibited the increase in the number of ATF3-IR positive cells induced by paclitaxel in DRG

**Figure 3:**
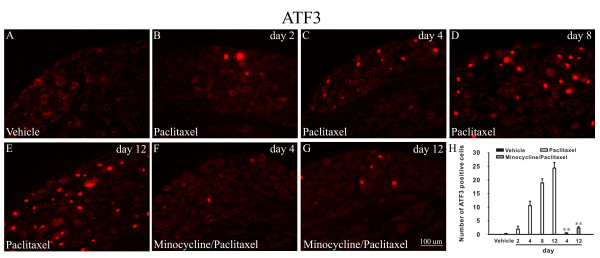
**Minocycline reduced up-regulation of ATF3 in DRG induced by paclitaxel**. No ATF3-positive cells in L4 DRG were observed in the vehicle-treated rats (A). Following application of paclitaxel, there is a progressive and significant increase in the number of ATF3 positive cells in DRG (B-E), while in minocycline/paclitaxel-treated rats, ATF3 positive cells were significantly decreased (F, G). Histogram representing the mean number of ATF3 positive cells under various treated conditions (H). **P < 0.01 compared with the corresponding time points of paclitaxel group (n = 5/group).

**Figure 4:**
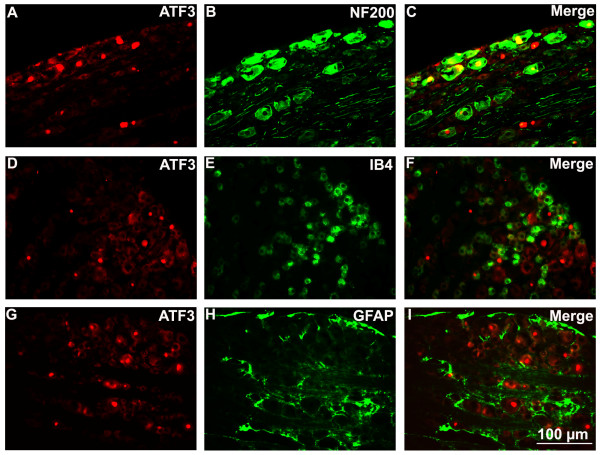
**Double immunofluorescence staining showed colocalization of activating transcription factor 3 (ATF3) with neurofilament 200 (NF200)**. Upregulated ATF3 induced by paclitaxel was co-localized with NF200-labeled cells (A-C), but not with IB4-labeled cells (D-F) or GFAP-labeled cells (G-I).

To assess whether the loss of IENF is accompanied by the sensory cell injury. We examined the levels of ATF3 in DRG at various time points. The result showed that paclitaxel treatment significantly increased the number of ATF3-immunoreactivity (IR) positive cells in L4 DRG (Figure 3B-E and 3H) compared with vehicle-treated rats in that ATF3-IR positive cells were hardly found (Figure 3A). Meanwhile, double immunofluorescence staining showed that ATF3 was co-localized with NF200-labeled cells (A fiber neuronal marker), but not with IB4-labeled cells (C fiber neuronal marker) or GFAP-labeled cells (satellite cell marker) (Figure 4). Furthermore, pretreatment with minocycline inhibited the increase of ATF3 (0.44 ± 0.18 and 1.33 ± 0.50 in minocycline/paclitaxel group versus 12.44 ± 1.68 and 24.33 ± 2.12 in paclitaxel group on day 4 and 12, respectively) (Figure 3F and 3G).

### Minocycline inhibited infiltration of macrophages induced by paclitaxel in DRG

**Figure 5:**
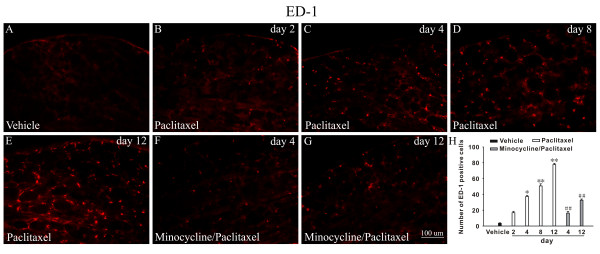
**Minocycline inhibited infiltration of macrophages into DRG induced by paclitaxel**. There is a significant increase on days 4, 8 and 12 in the number of ED-1 positive cells in L4 DRG after paclitaxel treatment (B-E), *P < 0.05;**P < 0.01 compared with the vehicle group. ED-1 positive cells in minocycline/paclitaxel-treated rats were significantly decreased compared with the corresponding time points of paclitaxel group, ##P < 0.01 (F, G). Histogram represents the mean number of ED-1 positive cells under various treated conditions (H) (n = 5/group).

Few macrophages stained by ED1 were detected in L4 DRG of vehicle treated rats (Figure 5A). However, following paclitaxel treatment, a significant increase in the number of macrophages was observed in DRG on day 2 (16.83 ± 1.81), 4 (37.33 ± 1.30), 8 (50.66 ± 2.66) and 12 (77.66 ± 1.62) (Figure 5B-E). Furthermore, the infiltration of macrophages induced by paclitaxel was significantly inhibited by minocycline treatment on day 4 (Figure 5F) and 12 (Figure 5G). Because recent study showed that the infiltration of macrophages in the spinal cord played a vital role in neuropathic pain [14], we also examined whether paclitaxel could induce the macrophage to infiltrate into the spinal cord. However, in our experiment, no obvious ED1-positive cells were detected in the spinal dorsal horn in either paclitaxel group or vehicle group (data not shown).

## Discussion

In the present study, we reported that moderate-dose paclitaxel induced mechanical allodynia, accompanied by partial loss of IENF in the hind paw glabrous skin, up-regulation of ATF3 and macrophages infiltration in DRG. Further results showed that minocycline, an inhibitor of microglia/macrophage activation, inhibited the loss of IENF as well as the increase of ATF3 and macrophages infiltration in DRG. Such inhibitory action of minocycline is parallel with its prevention of paclitaxel-induced allodynia. Our observations firstly implied that inhibition on loss of IENF and macrophages infiltration might contribute to the minocycline preventive effect on paclitaxel-induced allodynia.

### Impact of paclitaxel on IENF and macrophage

It is well established that paclitaxel treatment could elicit peripheral sensory neuropathy. Degeneration of nervous fibers has been currently suggested as the possible mechanism underlying the paclitaxel induced mechanical allodynia. It has been reported that application of low-dose paclitaxel (2 mg/kg) induced the loss of IENF [5,15]. In vitro study also showed that paclitaxel directly applied to the axonal resulted in degeneration of axons [16]. In our present study, moderate-dose paclitaxel (8 mg/kg) also significantly decreased the number of IENF. There are several explanations to such degeneration of fibers. For example, Nogales et al indicated that paclitaxel impaired axoplasmic transport by binding toβ-tubulin which has been thought as the cause resulting in degeneration of IENF [17]. While evidence against this hypothesis implied that paclitaxel directly impaired mitochondria function which might lead to degeneration of the fiber terminals [2].

We also found that paclitaxel induced the expression of ATF3 in DRG. It has been shown that ATF3 might have a survival/regenerative function in sensory neurons [18]. Evidence has shown that lack of target derived growth factors secondary to nerve injury resulted in the ATF3 up-regulation [19,20]. Therefore, in our study, it appeared that the decreased availability of target-derived growth factors due to the degeneration of IENF following paclitaxel administration induced expression of ATF3. In addition, double-staining showed that expression of ATF3 mainly focused on the NF200-positive cells, the result indicated that paclitaxel mainly induced the injury of Aβ-fiber neurons and is consistent with Dougherty's report that paclitaxel treatment in cancer patients impairs the Aβ fiber function [21].

In the present study, we also observed marked hyperplasia of macrophage in the DRG following application of paclitaxel. The result is consistent with Peter's report that intravenous infusion of high-dose paclitaxel induced hypertrophy and hyperplasia of macrophage in DRG and sciatic nerve [22]. The increased macrophages observed in the current study may be due to infiltration of macrophages into the DRG. This hypothesis is supported by our latest observation that application of moderate-dose paclitaxel elevated the level of chemotatic factor in DRG (unpublished data). Functionally, the activated macrophage may help remove degeneration neuronal debris and myelin following the peripheral nerve injury [23]; on the other hand, it may also contribute to the pathological pain through the release of proinflammatory cytokines which is capable of sensitizing primary afferent neurons [24,25].

### Role of Minocycline in degeneration of IENF and infiltration of macrophages induced by paclitaxel

Cata's study showed that minocycline, an inhibitor of microglia/macrophage activation, ameliorated taxol-induced hyperalgesia. It has been hypothesized that the immunomodulatory activity of minocycline underlies its protective effect on taxol-induced neuropathic pain. However, the exact mechanism is still unclear. In our present study, minocycline attenuated the loss of IENF, which was parallel with the reduced allodynia. It has been shown that minocycline decreased recruitment and activation of macrophage thereby slowing Wallerian degeneration [26]. In addition, minocycline treatment reduces oligodendrocyte death and attenuates axonal dieback after spinal cord injury [13]. Moreover, mitochondrial impairment, which has been suggested to contribute to degeneration of nerve fibers, could be prevented by minocycline. Therefore, it is possible that, by protecting the integrity of IENF, minocycline attenuated the loss of IENF induced by paclitaxel. This hypothesis was also supported by our present finding that minocycline decreased ATF3 up-regulation in DRG neurons.

Furthermore, we observed that paclitaxel-induced macrophages infiltration into DRG was obviously prevented in minocycline treated rats. Several lines of evidence proved that minocycline could inhibit the activation and migration of macrophages and reduce production of macrophage proinflammatory factors [27,28] which mediated peripheral nerve degeneration [28]. Furthermore, inhibition of macrophage responses might prevent nerve fiber degeneration by prohibiting the phagocytosis of axon ends [13,28]. Although in the present study, activation of macrophages around the peripheral nerve fibers was not examined, its destructive effect on the IENF could not be excluded. Therefore, inhibition of macrophage responses might contribute to minocycline preventive effect on IENF loss induced by paclitaxel. However, additional studies are needed to elucidate the role of minocycline in protecting IENF from paclitaxel-induced injury.

## Conclusions

In conclusion, our present data provided evidence that allodynia evoked by moderate-dose paclitaxel might be associated with the degeneration of IENF and recruitment of macrophages. Importantly, inhibition of minocycline on paclitaxel-induced allodynia might be mediated by interruption of loss of IENF and macrophage responses. Minocycline, a well-tolerated and widely used clinical drug, might represent a potential agent for preventing paclitaxel-induced neuropathic pain and subsequently improving patients' outcome.

## Methods

### Experimental Animals

Male Sprague-Dawley rats weighting 220-280 g were purchased from the laboratory animal center of Sun Yat-Sen University, the permit number for this study is SCXK(Guangdong)2009-0011. All animal experimental procedures were approved by the Sun Yat-Sen University Animal Care and Use Committee and were carried out in accordance with the guideline of the National Institutes of Health on animal care and the ethical guidelines. The minimum number of animals was used in each experiment, and in all cases every effort was made to minimize any pain and suffering in the subject animals.

### Drug administration

Paclitaxel (Taxol, Bristol-Myers Squibb, 6 mg/ml) was diluted with saline (1:3) and injected i.p (cumulative dose of 24 mg/kg) on 3 alternate days (days 1, 4 and 7)[29]. Minocycline (Sigma) was administered (i.p) daily at a dosage of 30 mg/kg and begin prior one day to the paclitaxel [30]. Rats received minocycline always 30 min before application of paclitaxel and continued up to eight days. Minocycline was dissolved in saline and stored in solution at 200 mg/ml and then diluted in saline before the administration.

### Behavioral testing

The rats were accommodated to the testing environment by placement within testing chambers for 15-20 minutes on the three separate days just prior to drug administration. Mechanical sensitivity was assessed using von Frey hairs as described previously [12]. Briefly, rats were placed under three different transparent Plexiglas chambers positioned on a wire mesh floor. Fifteen minutes were allowed for habituation. Each stimulus consisted of a 2-3s application of the von Frey hair to the middle of plantar surface of the foot with 5 min interval between stimuli. Brisk withdrawal or licking of the paw following the stimulus was considered a positive response. The experimenter who conducted the behavioral tests was blinded to all treatments.

### Immunohistochemistry

All rats used in the immunohistochemistry experiments had confirmed to have a characteristic of behavior. Rats were deeply anesthetized with urethane (1.5 mg/kg, i.p.) at different time points, the chest was opened, and then quickly perfused through the ascending aorta with a warm heparinized saline, followed by 4% paraformaldehyde in 0.1 M phosphate buffer, pH 7.2-7.4, 4°C. The glabrous skin of hind paw and the L4 DRG was excised, post-fixed overnight and cryoprotected for 24 h in 30% sucrose in PB. Cryostat sections (16 μm) were cut and processed for immunohistochemical staining as previously described [31,32]. Sections were blocked with 3% donkey serum in 0.3% Triton X-100 for 1 hour at the room temperature, and then incubated overnight at 4°C with rabbit anti-protein gene product 9.5 primary antibody (PGP9.5, 1:2000, Chemicon) for skin or rabbit anti-ATF3 antibody (1:200, Santa Cruz) or mouse anti-Macrophage antibody (ED-1, 1:200, Chemicon) for DRG. For double staining, the sections were incubated with rabbit anti-ATF3 antibody (1:200, Santa Cruz) and mouse anti-neurofilament-200, an A-fiber neuronal marker (NF200, 1:300, Chemicon), mouse anti-isolectin B4, a C-fiber neuronal marker (IB4, 1:200, Sigma) or glial fibrillary acidic protein, a satellite cell marker (mouse anti-GFAP, 1:2000, Chemicon). After rinsing three times with PBS, sections were incubated in donkey anti-rabbit IgG secondary antibody labeled with Cy3 (1:500, Jackson) or a mixture of IgG secondary antibody labeled with Cy3 and FITC respectively (1:500, Jackson) for 1 h at a room temperature. Five rats were included for each group for immunohistochemistry quantification. Three DRG tissue sections per animal are randomly selected, the number of ATF3 or ED-1 positive cells was examined with a Leica (Leica, Germany) fluorescence microscope and images were captured with a Leica DFC350 FX camera. For IENF quantification, we selected five plantar skin sections per animal and chose three sights for each section randomly. Images of immunohistochemical results were obtained using an Zeiss LSM710 confocal microscope and analyzed with a Bitplane Imaris V6.4. All ascending nerve fibers that were seen to cross into the epidermis were counted, no minimum length was required and fibers that branched within the epidermis were counted as one. The number of IENF per sight was counted. To confirm the specificity of the primary antibody, control sections were incubated without primary antiserum.

### Statistical analysis

Blinded evaluator analyzed all images. The number of the fibers, ATF3 or ED1 positive cells every sight was expressed as mean ± SEM. Data were compared with student's t-test, *P *< 0.05 was considered significant. For behavioral experiments, one-way ANOVA followed by post hoc test was used and *P *< 0.05 was considered significant.

## Abbreviations

IENF: intraepidermal nerve fiber; ATF3: activating transcription factor 3; DRG: dorsal root ganglion; TRPV4: transient receptor potential vanilloid 4

## Competing interests

The authors declare that they have no competing interests.

## Authors' contributions

CCL and TY carried out all the experiment and drafted the manuscript. NL and YC participated in the design of the study. ZQZ conceived of the study, and participated in the design and helped to draft the manuscript. XGL and WJX coordinated and supervised the experiments, analyzed the data and wrote the manuscript. All authors read and approved the final manuscript.
